# Updates in Therapy for Advanced Melanoma

**DOI:** 10.3390/cancers8010017

**Published:** 2016-01-15

**Authors:** Bhavana P. Singh, April K. S. Salama

**Affiliations:** 1 Department of Internal Medicine, Duke University Medical Center, Durham, NC 27710, USA; bhavana.pendurthi@dm.duke.edu; 2 Division of Medical Oncology, Department of Internal Medicine, Duke University Medical Center, Durham, NC 27710, USA

**Keywords:** melanoma, immunotherapy, targeted therapy, CTLA-4, PD-1, BRAF, MEK

## Abstract

Cutaneous melanoma is one of the most aggressive forms of skin cancer, and is correlated with a large proportion of skin cancer-related deaths. Therapy for cutaneous melanoma has advanced greatly through careful identification of therapeutic targets and the development of novel immunotherapeutic approaches. The identification of BRAF as well as other driver mutations, have allowed for a specialized approach to treatment. In addition, immune checkpoint inhibition has dramatically changed the treatment landscape over the past 5–10 years. The successful targeting of CTLA-4, as well as PD-1/PD-L1, has been translated into meaningful clinical benefit for patients, with multiple other potential agents in development. Systemic therapy for cutaneous melanoma is becoming more nuanced and often takes a multifaceted strategy. This review aims to discuss the benefits and limitations of current therapies in systemic melanoma treatment as well as areas of future development.

## 1. Background

Melanoma has long been recognized as a potentially aggressive form of skin cancer. The incidence of melanoma in the United States is projected to be approximately 73,870 new cases in 2015 [1]. Although melanoma is much less common than other cutaneous malignancies, such as basal cell and squamous cell carcinoma, it accounts for the majority of skin cancer related deaths [2]. There is a large variance in survival rates depending on the extent of disease, with early stage melanoma often being cured by surgery alone, while the historic five-year survival rate for metastatic disease is only 16.6% [2]. Fortunately, the previously dismal prognosis of this disease is evolving with recent advances in systemic therapy.

The identification of BRAF as well as other driver mutations such as KIT, have allowed for a different approach to systemic therapy in a selected subset of patients. Targeted therapies, including selective BRAF and MEK inhibitors, have improved rates of progression-free and overall survival in patients whose melanoma harbors a *BRAF* V600 mutation [3,4,5,6]. The landscape of therapeutics for melanoma was also revolutionized with the discovery of a new class of immune modulators, first with the immune checkpoint inhibitor ipilimumab, and more recently with anti PD-1 antibodies, which have shown increased overall survival in recent trials [7,8,9]. Other strategies including novel combinations, in addition to adoptive cell therapy and viral therapy continue to be studied.

Overall, these advancements have lead to growing optimism within the field and have transformed the way this disease is treated clinically. This review focuses on the benefits and limitations of current therapies in the management of advanced melanoma as well as areas of future development.

### A Historical Perspective

Before 2010, systemic treatment for locally advanced melanoma outside of clinical trials was largely limited to cytotoxic chemotherapy and more traditional forms of immunotherapy. While small subsets of patients benefited from agents such as dacarbazine and temozolomide, the responses were often brief and a clear benefit had not been demonstrated in phase III trials [10,11]. High-dose interleukin-2 (IL-2) was approved for the treatment of metastatic melanoma based on data demonstrating sustained remissions in approximately 5%–10% of patients [12]. Many patients are not candidates for this type therapy given the substantial toxicity profile and the need to be administered at specialized centers. However, with the potential for a durable response that can last for decades, IL-2 remains an option for a selected subset of patients [13].

## 2. Targeted Therapy in Melanoma

### 2.1. BRAF

**Figure 1:**
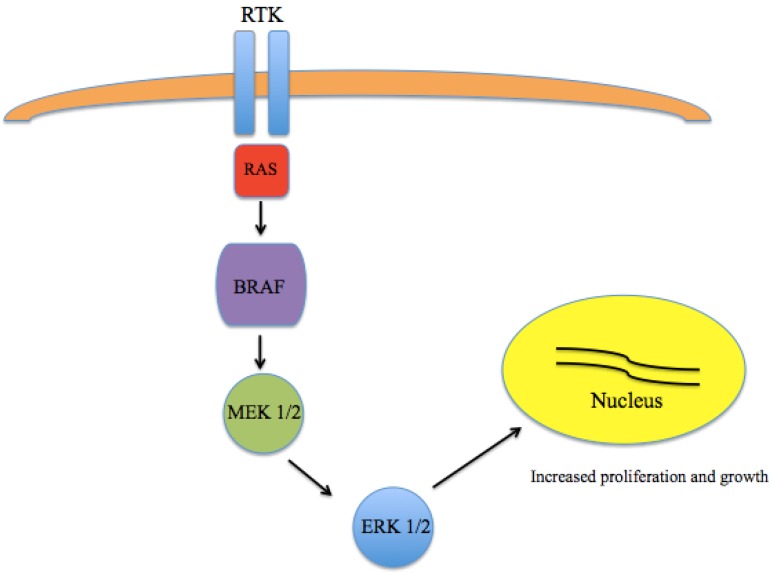
Mitogen Activated Protein Kinase (MAPK) pathway signaling. The RAS-RAF-MEK-ERK pathway transmits signals from extracellular ligands to specific intracellular effectors. Mutations in BRAF results in a constitutively active kinase leading to unregulated growth and proliferation.

A major breakthrough in the understanding of the pathogenesis of melanoma came with the discovery that a large number of melanomas harbor activating mutations in *BRAF* [14,15,16]. The most common mutation is in the amino acid position 600 of the *BRAF* gene (V600) [17]. While V600E appears to be the most common in melanoma, other variants, including V600K are also seen. These somatic missense mutations have been identified in up to 66% of malignant melanomas. As part of the RAS-RAF-MEK-ERK pathway, which normally serves to transmit signals from extracellular ligands to specific intracellular effectors, mutated *BRAF* results in a constitutively-active kinase leading to unregulated growth and proliferation (Figure 1).

Testing for the presence of a BRAF mutation should be considered in all patients with metastatic disease. Several methodologies have been used, with the most common being the usage of polymerase chain reaction (PCR) based assays, although recent data has demonstrated the feasibility of immunohistochemistry (IHC) as well. PCR-based companion diagnostics were approved in conjunction with vemurafenib and dabrafenib/trametinib in the United States, which include the cobas 4800 BRAF V600 (Roche Molecular Systems, Inc., Pleasanton, CA, USA, accessed 19 August 2015) and THxID™ BRAF (bioMérieux Inc., Marcy-l'Etoile, France, accessed 19 August 2015) tests, respectively [18,19]. Both assays appear to have a high level of agreement for detecting the presence of V600E mutations, with somewhat less sensitivity and specificity for other variants, including V600K. One potential disadvantage, however, is the length of time that may be needed to obtain a result from DNA based assays. IHC has also been evaluated as an alternative, and several studies evaluated a BRAF^V600E^ specific antibody, VE1 [20,21,22]. IHC was shown to be feasible, and demonstrated high sensitivity and specificity for the *BRAF^V600E^* mutation. It should be noted, however, that other BRAF^V600^ variants were not detected with this antibody.

Clinically, the development of vemurafenib and dabrafenib, highly selective BRAF^V600^ inhibitors, represented a major breakthrough in the treatment of metastatic melanoma. Promising clinical activity was seen in a phase I trial of vemurafenib, which demonstrated a response rate (RR) of 81% in the extension cohort of 32 melanoma patients [23]. Confirmation of this benefit was subsequently seen in phase II and phase III trials. In 675 patients that were randomly assigned to receive vemurafenib *versus* dacarbazine, the RR in the vemurafenib arm was 48%, compared with 5% for those that received dacarbazine [3]. In an updated analysis that included extended follow up, a median overall survival (OS) of 13.6 months was seen in the vemurafenib group compared to 9.7 months in those in the dacarbazine arm [24]. Dabrafenib has also shown similarly promising results and was subsequently approved by the FDA in 2013. In a randomized phase III trial that included patients with previously untreated *BRAF*^V600^ stage IV melanoma, patients in the dabrafenib arm had an improved progression free survival (PFS) of 5.1 months compared to 2.7 months for dacarbazine [25]. In a more recent update, median OS in the dabrafenib group was 20 months, compared to 15.6 months in the dacarbazine arm, though survival analyses are confounded by the large percentage of patients who crossed over to the dabrafenib arm [26,27]. Despite the success of BRAF directed therapy, the development of resistance remains a major issue in most patients. While the mechanisms are likely multifactorial, MAPK pathway reactivation appears to play a major role and continues to fuel the need for the development of novel therapies [28,29].

### 2.2. MEK

Early attempts at targeting MEK were largely limited by toxicity, as well as limited antitumor activity [30]. Newer generation MEK inhibitors such as selumetinib, trametinib, cobimetinib, and binimetinib (MEK162) have shown promise, and have primarily been developed as part of a combination strategy along with BRAF inhibitors. As monotherapy, trametinib demonstrated a survival advantage compared with conventional chemotherapy [31]. When trametinib was compared with chemotherapy (DTIC or paclitaxel) in 322 patients with *BRAF*-mutated melanoma, median PFS and 6 month OS rates were greater in the trametinib group, at 4.8 months and 81%, *versus* 1.5 months and 67% in the chemotherapy group [31]. Binimetinib has also shown similar clinical efficacy in *BRAF*-mutant melanoma in a phase II study, as well as evidence of activity in *NRAS* mutated disease [32]. Selumetinib has demonstrated modest clinical activity in patients with metastatic uveal melanoma, with an improvement in PFS when compared to chemotherapy [33]. However, with overall response rates that are lower than BRAF targeted therapies, the major focus of MEK targeted therapy continues to be as an integral part of a combination strategy in *BRAF* mutated disease.

### 2.3. KIT

In recent years, KIT has also been a target of interest in advanced melanoma, as certain subsets of patients appear to harbor activating mutations, predominantly acral and mucosal subtypes [34]. The most common mutations are *KIT^L576P^* on exon 11 and *KIT^K642E^* on exon 13, although others have been reported in case series [35]. A few phase II trials have investigated the efficacy of imatinib for melanomas with an alteration in KIT. In one study, patients were included if their tumors carried a mutation or amplification in KIT and of the 43 patients enrolled, 16 patients had single mutations in exon 11 and six patients had a single mutation in exon 13. 14 patients showed mutations in other exons and five patients had multiple *KIT* mutations. For the entire population of 43 patients, 23.3% had a partial response (PR), stable disease (SD) was observed in 30.2% and progressive disease was seen in 46.5% of patients [36]. Notably, nine of the 10 PRs were seen in patients with an exon 11 or 13 mutation, suggesting these mutations are a more reliable predictor of response. A study of 28 patients treated with imatinib demonstrated an overall RR of 16%, which was defined as responses lasting more than one year [37]. Again, in this study clinical benefit appeared to be largely confined to patients whose disease harbored a mutation in exon 11 or 13. Another study in this patient population confirmed this pattern of potential benefit in selected KIT mutations [38]. Additionally, a more recent study has also demonstrated potential clinical activity with nilotinib. In this study, patients who were intolerant or whose disease had progressed after imatinib therapy (cohort A), as well as those with brain metastases (cohort B) were treated with nilotinib 400 mg twice daily [39]. A total of 11 patients were treated in cohort A, and two had a PR, while no responses were seen in the eight patients treated in cohort B. For the total study population, the median time to progression was 3.3 months, and OS was 9.1 months. While predictors of response to KIT targeted therapy continue to be refined, these agents represent a potential treatment option for selected subsets of patients.

## 3. Immune Checkpoint Inhibition

### 3.1. CTLA-4

**Figure 2:**
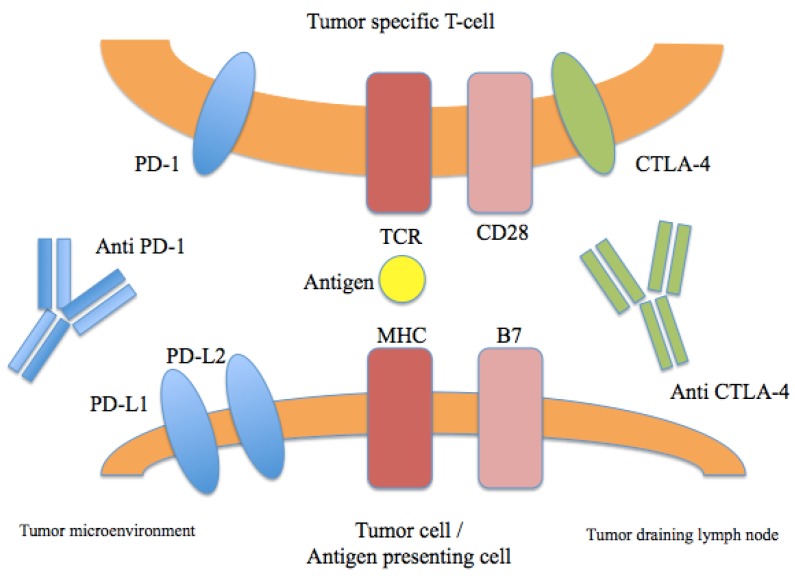
Immune checkpoints in melanoma therapy. Blockade of CTLA-4 or PD-1/PD-L1 results in the activation of T cells with specificity for cancer cells.

In addition to the development of BRAF and MEK inhibitors, breakthroughs in the field of immunotherapy have also dramatically impacted the treatment landscape for advanced melanoma. Ipilimumab is a fully human IgG1 monoclonal antibody that blocks cytotoxic lymphocyte associated antigen-4 (CTLA-4), a coinhibitory receptor that regulates T-cell activation and the function of T-regulatory cells (Figure 2). It was the first agent to demonstrate an improvement in overall survival in a randomized phase III trial in advanced melanoma [7]. In this landmark study, previously treated patients with advanced melanoma were randomized to receive ipilimumab either with or without a peptide vaccine. Interestingly, a cohort of patients receiving ipilimumab appeared to derive long-term benefit and demonstrated a prolonged treatment response. The potential for durable benefit was also seen in another study, in which previously untreated patients received ipilimumab with or without dacarbazine. In this trial, patients who received ipilimumab demonstrated higher rates of survival at one, two, and three years [40]. A recent follow up analysis of this study demonstrated a five-year survival rate of 18.2% in the ipilimumab plus dacarbazine arm *versus* 8.8% in the placebo plus dacarbazine group (*p* = 0.002) [41]. Additionally, another analysis of 1861 melanoma patients treated with ipilimumab across multiple studies showed a three-year OS rate of approximately 20%, with very few recurrences after that time [42]. These collective data demonstrate the potential for a durable survival benefit in a subset of advanced melanoma patients treated with ipilimumab.

### 3.2. PD-1

The programmed death 1 (PD-1) pathway is involved in immune regulation, mediated by binding of PD-1 and its ligands PD-L1/PD-L2 (Figure 2) [43]. The development of PD-1 blocking antibodies has now shown clinical benefit in melanoma as well a wide variety of tumor types [44]. Most recently, pembrolizumab and nivolumab received regulatory approval for the treatment of ipilimumab-refractory melanoma, though a number of agents targeting this pathway are at various phases of clinical development. In a phase I study of nivolumab, a fully human IgG4 antibody, conducted in 296 patients, the cumulative RR was 28% among patients with melanoma [44]. Overall, nivolumab appeared to be very well tolerated, and common immune-related adverse events were generally mild and included rash, diarrhea, and pruritus. In a cohort expansion that included 107 patients with melanoma, median OS was 16.8 months, and one- and two-year survival rates were 62% and 43%, respectively [45]. Confirmation of this benefit was seen in a randomized phase III study, in which nivolumab was associated with an improvement in OS compared with dacarbazine in patients with previously untreated melanoma [46]. The RR was 40% with nivolumab compared with 13.9% in the dacarbazine group. Nivolumab has also demonstrated a benefit in patients who have progressed on prior ipilumumab. In a randomized phase III study comparing nivolumab to chemotherapy, the RR was 32% in the nivolumab arm, *versus* 6% in the chemotherapy arm [9]. The safety profile was consistent with prior studies, with a relatively low rate of grade 3-4 adverse events.

Pembrolizumab is a humanized anti-PD-1 IgG4 antibody that has also demonstrated a clinical benefit in patients with advanced melanoma, as well as other malignancies. An initial dose escalation study in 135 patients with metastatic melanoma that included both ipilimumab-naive patients and those with progression on prior treatment with ipilimumab, demonstrated a RR of 38% across all cohorts, with patients on the highest dose of pembrolizumab (10 mg/kg every two weeks) showing a RR of 52% [47]. Overall, pembrolizumab appeared to be well tolerated; grade 3/4 drug-related adverse events occurred in only 13% of patients, with the highest rate seen in the cohort that received 10 mg/kg every two weeks. To further investigate the optimal dosing of pembrolizumab in this phase I study, a cohort of ipilimumab refractory melanoma patients were randomized to receive pembrolizumab at 2 mg/kg or 10 mg/kg every three weeks [48]. A total of 173 patients were treated, and the RR was 26% in both arms. Consistent with prior data, pembrolizumab appeared to be well tolerated, with comparable safety profiles irrespective of dose. In the first line setting, randomized phase III data has also confirmed the benefit of pembrolizumab in advanced melanoma [49]. In this study of 834 patients, subjects were assigned to receive pembrolizumab 10 mg/kg every two or three weeks or ipilimumab at 3 mg/kg for four doses. Response rates in both pembrolizumab arms were 33.7% and 32.9%, which corresponded with every two and three weeks, respectively. This was significantly increased when compared with ipilimumab, which had a RR of 11.9%. The study also demonstrated an improvement in six-month PFS rates, with 47.3% for pembrolizumab every two weeks, 46.4% for pembrolizumab every three weeks, *versus* 26.5% for ipilimumab. Additionally, less severe treatment related adverse events were seen in the pembrolizumab arms. Importantly, an updated analysis of the initial phase I study highlighting the potential for durable clinical benefit was recently presented [50]. In the total study population, consisting of 655 patients across multiple cohorts, the overall RR was 33%, with a median duration of response of 28 months, further confirming the potential for durable benefit.

A number of other agents targeting the PD-1/PD-L1 pathway are at various phases of clinical development. Pidilizumab (CT-011), a humanized anti-PD-1 IgG1 antibody, was studied in a phase I dose escalation study of 17 patients with hematologic malignancies [51]. One patient with follicular lymphoma had a complete response, and another four patients demonstrated stabilization of disease for several months. However, in another study of 103 patients with metastatic melanoma who were randomized to receive two different doses of pidilizumab, the RR was low and the OS rate at 12 months was 64.5% [52].

Antibodies targeted against PD-L1 are also being pursued in melanoma and other tumor types, including BMS-936559, MPDL3280A (atezolizumab), MEDI4736 (durvalumab), and MSB0010718c (avelumab) [53,54]. In the initial phase I study of BMS-936559, a high-affinity human antibody, that included 52 patients with melanoma, nine patients demonstrated a response, including three with a complete response [55]. In this population, 14 of the 52 patients experienced stabilization of disease for greater than 24 weeks. MPDL3280A, an engineered anti PD-L1 antibody, has also demonstrated activity in patients with metastatic melanoma. In a phase I study that included an expansion cohort of 45 melanoma patients, the overall RR was 28% [56]. The activity of durvalumab, a fully human anti-PD-L1 IgG1 antibody, has also been explored in early phase studies that have included small cohorts of melanoma patients. Overall, the agent appears to be well tolerated with preliminary evidence of clinical benefit [55,57,58]. Lastly, avelumab, fully human anti-PD-L1 IgG1 antibody, is being developed across multiple tumor types, primarily outside of melanoma, including a planned trial for advanced Merkel cell carcinoma (NCT02155647).

## 4. Adoptive Cell Immunotherapy

Adoptive cell therapy (ACT) refers to the process of administering autologous or allogeneic tumor-reactive T or NK cells to patients with the intent of achieving tumor regression. This process occurs through the isolation of lymphocytes with high affinity for tumor antigens, which can be selected *ex vivo*, stimulated, expanded, and infused back into the patient and represents an area of great promise in the treatment of metastatic melanoma [59]. In melanoma, it has been shown that from an excised tumor, numerous tumor antigen-specific T cells can be isolated [60]. In one study, cell therapy with tumor infiltrating lymphocytes (TIL) was reported to result in an objective response rate of around 49% with twenty of the 93 patients (22%) achieving complete tumor regression [61]. Of note, TIL together with high-dose IL-2 has consistently demonstrated durable clinical response rates near 50% or more in multiple clinical trials [62,63,64]. Limitations of this approach is the potential logistical and technical hurdles from patient selection, tumor resection, and expansion of adequate numbers of viable TILs culture [65]. To address some of these, novel strategies, such as genetically modified T cells are being developed. Some tumor-associated antigens have been identified, including melanoma antigen recognized by T cells 1 (MART-1) and cancer testis antigen (NY-ESO-1) [66,67]. In a broader cohort of patients with metastatic cancer, not specifically melanoma alone, 5 of nine patients demonstrated cancer regression using RECIST criteria following infusion of anti-MAGE-A3 TCR gene-engineered T cells [68,69]. These studies have shown the promise of TIL in the management of metastatic melanoma.

## 5. Combination Therapy

Combination approaches to therapy provide a rational strategy to potentially overcome resistance, and have shown a great deal of promise in melanoma. While toxicity remains a concern in some instances, the field continues to rapidly evolve and it is likely to become a mainstay of future therapeutic approaches.

### 5.1. Targeted Therapies

One key strategy has been the simultaneous inhibition of both BRAF and MEK, as summarized in Table 1, which is based on data from preclinical studies that have shown that dual BRAF and MEK inhibition increases apoptosis and delays the onset of resistance compared to BRAF inhibitors alone [70,71]. Furthermore, a common mechanism of resistance to BRAF inhibitors is reactivation of the MAPK pathway. For this reason, it was hypothesized that BRAF inhibitors combined with MEK inhibitors would potentially overcome such resistance [72]. Several studies have assessed the efficacy and toxicity of concurrent administration of BRAF and MEK inhibitors, including a number of randomized phase III trials. One of the earliest studies proved the combination of dabrafenib and trametinib to be feasible, and a subsequent randomized phase II trial compared the use of dabrafenib plus trametinib (at either 1 or 2 mg) with dabrafenib alone [70]. In this study of 162 patients with *BRAF* V600 mutated metastatic melanoma, the combination of dabrafenib and trametinib at 2 mg had an improved median PFS at 9.4 months, compared with 5.8 months in the dabrafenib monotherapy group. Clinically, while the rate of pyrexia was increased with combination therapy, there appeared to be a reduction in the rate of BRAF inhibitor-related hyper proliferative skin lesions, consistent with the observation that these result from paradoxical activation of the MAPK pathway in BRAF wild type cells [73]. Two additional randomized phase III studies also demonstrated clinical benefit of the combination when compared to a single agent dabrafenib or vemurafenib [5,6]. A recently updated analysis of the comparison of dabrafenib plus trametinib *versus* dabrafenib alone confirmed these findings, with an improvement in median OS in the combination arm (25.1 months *versus* 18.7 months) [74]. Recently presented data also demonstrated that the addition of cobimetinib to vemurafenib was associated with a significant improvement in PFS among patients with *BRAF* V600 mutated metastatic melanoma. In this randomized trial of 495 patients with locally advanced or metastatic disease, those who received vemurafenib plus cobimetinib had an improved PFS, with 9.9 months in the combination arm *versus* 6.2 months in the vemurafenib alone arm. Overall survival at nine months was also improved with dual BRAF/MEK therapy, at 81% compared to 73% in the control group [4]. In an updated analysis, median PFS in the combination arm was 12.3 months *versus* 7.2 months in the vemurafenib plus placebo group [75]. Interestingly, correlative analyses from this trial suggested that a subset of patients may have additional oncogenic mutations, including those in the RAS/RAF pathway, highlighting the potential for additional combination strategies. The combination of the BRAF inhibitor encorafenib with the MEK inhibitor binimetinib has also shown promise. Data from a phase I/II study demonstrated high overall response rates consistent with prior experience, and a potentially favorable toxicity profile with lower rates of pyrexia and photosensitivity than reported with other combinations [76]. Taken collectively, the body of data demonstrating improved response rates, meaningful improvements in PFS and OS, along with a manageable toxicity profile, establishes dual BRAF/MEK inhibition as a standard of care option for melanoma patients whose tumors harbor a *BRAF* V600 mutation. Efficacy amongst targeted therapy combinations appears similar, though no prospective trials have been conducted directly comparing different agents. A number of toxicities appear to be a class effect, though there are some potential differences that are notable and more common with certain agents. Among these include higher rates of febrile reactions with dabrafenib and trametinib, and photosensitivity with vemurafenib. Interestingly, the combination of binimetinib and encorafenib appeared to have lower rates of both pyrexia and photosensitivity, though larger studies are ongoing.

**Table 1:** BRAF + MEK combination studies.

Study	Trial Design	Agents Studied	N	RR (%)	Median PFS (Months)	OS (%)
*Dabrafenib + trametinib studies*						
Robert *et al.* [5]	Randomized, phase III	Dabrafenib + trametinib	352	64	11.4	72 (12 months)
		Vemurafenib	352	51	7.3	65 (12 months)
Long *et al.* [6,74]	Randomized, phase III	Dabrafenib + trametinib	211	67	11	93 (9 months)
		Dabrafenib + placebo	212	51	8.8	85 (9 months)
Daud *et al.* [77]	Randomized, phase I—II	Dabrafenib + trametinib (150/2)	54	76	9.4	51 (24 months)
Flaherty *et al.* [70]						
		Dabrafenib + trametinib (150/1)	54	50	NR ^a^	NR
		Dabrafenib (150)	54	54	5.8	44 (24 months)
*Vemurafenib + cobimetinib studies*						
Larkin et al. [4,75]	Randomized, phase III	Vemurafenib + cobimetinib (960/60)	247	70	12.3	81 (9 months)
		Vemurafenib (960) + placebo	248	50	7.2	73 (9 months)
*Encorafenib + binimetinib studies*						
Sullivan *et al.* [76]	Randomized, phase II	Encorafenib + binimetinib (600/45)	38	72	11.3	NR
					(all doses combined)	
		Encorafenib + binimetinib (400/45)	4	78		NR

^a^ NR: not reported.

### 5.2. Immunotherapy Combinations

Clinically, both CTLA-4 and PD-1 directed monotherapy have proven benefit in advanced melanoma. Additionally, in preclinical mouse models, the combination of CTLA-4 and PD-1 blockade appeared to be synergistic, leading to the clinical development of this combination [78]. .Correlative analyses in patients treated with dual immune checkpoint blockade suggests that CTLA-4 and PD-1 blockade have distinct, non overlapping immunomodulatory effects, further supporting this combination strategy [79]. In a phase I study which included a cohort of 53 patients treated with ipilimumab and nivolumab, substantial clinical activity was seen [80]. Forty percent of the patients had an objective RR, with 16 patients experiencing a reduction of 80% or more in tumor volume. In a subsequent randomized phase II study of 142 previously untreated patients comparing concurrent therapy *versus* ipilimumab alone, those in the combination arm had an objective response rate of 59% *versus* 11% in the group that received ipilimumab [81]. Recently reported results from a three-arm phase III study of 945 previously untreated patients with advanced melanoma confirmed these results. In this study, patients were randomized to receive nivolumab alone, nivolumab plus ipilimumab, or ipilimumab alone. Response rates were similar to those reported in other studies, with a RR of 44% and 19% for nivolumab and ipilimumab monotherapy, respectively. The combination resulted in an improvement in response, (58%) as well as an improvement in progression free survival compared to the monotherapy arms, with a median PFS of 11.5 months *versus* 6.9 months with nivolumab and 2.9 months with ipilimumab [82]. Subgroup analyses suggested that the cohort of patients with PD-L1 negative tumors potentially derived the most benefit from combination blockade, though additional follow up is warranted. There was an increase in the rate of toxicities seen with this combination, with up to 50% grade 3/4 adverse events, although most appear to be treatable and reversible with prompt intervention. Key results from these studies are summarized in Table 2.

**Table 2:** Selected PD-1 combinations.

Study	Trial Design	Agents Studied	N	RR (%)	PFS (Months)	OS (%)
Wolchok *et al.* [83]	Phase I	Nivolumab + ipilimumab	314	57.6	11.5	75 (2 years)
Sznol *et al.* [84]	multiple cohort					
		Nivolumab	316	43.7	6.9	NC ^a^
		Ipilimumab	315	19	2.9	NC
Hodi *et al.* [85],	Randomized, phase II	Ipilimumab + nivolumab	72 *	60	8.9	NR ^b^
Postow *et al.* [81]						
		Ipilimumab + placebo	37 *	11	4.7	NR
Larkin *et al.* [82]	Randomized, phase III	Ipilimumab + nivolumab	314	57.6	11.5	NR
		Ipilimumab monotherapy	315	19	2.9	NR
		Nivolumab monotherapy	316	43.7	6.9	NR

^a^ NC: not calculated, ^b^ NR: not reported, * BRAF wild type patients.

### 5.3. Future Opportunities for Combination Therapy

Recent years have seen dramatic advances in systemic therapy for advanced melanoma and future approaches will likely focus on minimizing toxicity while maximizing clinical benefit. In addition to novel immunomodulatory targets, such as LAG-3, in which the monoclonal antibody BMS-986016 is currently being tested in combination with nivolumab (NCT01968109), as well as CD40, in which CP-870,893 recently showed some activity in combination with tremelimumab, other strategies are also being pursued [86]. Talimogene laherparepvec (T-VEC), a herpes simplex virus-1 (HSV) oncolytic vaccine, has demonstrated potential for durable responses in patients with unresectable melanoma, particularly in patients with soft tissue or nodal disease. Overall T-VEC appears to be fairly well tolerated, with low rates of grade 3/4 adverse events [87]. Data from a combination study with ipilimumab in 18 patients with unresectable melanoma showed that the combination of ipilimumab plus T-VEC appeared to be safe. Clinical activity was encouraging, with a RR of 56%, many of which appeared to be durable [88]. A trial is currently ongoing comparing the combination of T-VEC plus pembrolizumab *versus* pembrolizumab alone (NCT02263508). Additionally, the combination of targeted therapies plus immunotherapies may hold promise as new agents continue to be developed. A body of preclinical and correlative data suggests that selective inhibition of BRAF may have a number of immunomodulatory effects, including in enhanced T cell recognition and melanoma antigen expression, as well as increased T cell infiltration in the tumor supporting a rationale for combined therapy [89,90,91]. While concurrent vemurafenib and ipilimumab was shown not to be feasible due to hepatotoxicity, sequential administration was shown to be safe, with some potential for efficacy [92,93]. With newer immunotherapies now available, additional combinations are being pursued and selected ongoing trials are highlighted in Table 3.

**Table 3:** Selected ongoing combination studies in melanoma.

Combination	Study Population	Status	Study Design
Nivolumab + ipilimumab (NCT02320058)	Patients with melanoma brain metastases	Recruiting	Single arm phase II
Pembrolizumab + pegylated IFN alfa-2b and pembrolizumab + ipilimumab (NCT02089685)	Advanced/unresectable or metastatic melanoma or renal cell carcinoma	Recruiting	Single arm phase I Randomized expansion cohorts
Ipilimumab ± T-VEC (NCT01740297)	Advanced/unresectable melanoma, with injectable tumor	Recruiting	Phase Ib, II
Pembrolizumab + T-VEC (NCT02263508)	Advanced/unresectable melanoma, with injectable tumor	Active but not recruiting	Phase Ib/III
Ipilimumab + nivolumab and dabrafenib + trametinib (NCT02224781)	Advanced/unresectable melanoma, BRAF mutated	Recruiting	Randomized phase III, comparing sequence
Ipilimumab ± dabrafenib ± trametinib (NCT01940809)	Unresectable or metastatic malignant melanoma, BRAF mutated	Recruiting	Phase I
Pembrolizumab + trametinib and dabrafenib (NCT02130466)	Advanced (unresectable Stage III) or metastatic (Stage IV) melanoma	Recruiting	Phase II/III
MPDL3280A + vemurafenib or vemurafenib + cobimetinib (NCT01656642)	Metastatic melanoma, with BRAFV600 mutation	Recruiting	Phase II
MEDI4736 + dabrafenib and trametinib or with trametinib alone (NCT02027961)	Stage IIIc (unresectable) or Stage IV (metastatic) melanoma	Recruiting	Phase II/III

## 6. Conclusions

Effective targeted treatments and new breakthroughs in immunotherapy have been shown to improve survival and are now a part of the routine clinical care of melanoma patients. The challenge for clinicians and researchers moving forward will be to determine the optimal tools for patient selection, sequencing of therapy, and ideal combination strategies. The translation of key scientific findings into well-designed clinical studies will be critical in answering the most important questions in the future. Overall there has been much progress in the development of systemic therapy for advanced melanoma, which will hopefully benefit many patients to come.
